# Association between tea consumption and osteoporosis: A meta-analysis

**DOI:** 10.1097/MD.0000000000009034

**Published:** 2017-12-08

**Authors:** Kang Sun, Le Wang, Qingping Ma, Qiaoyun Cui, Qianru Lv, Wenzheng Zhang, Xinghui Li

**Affiliations:** a Tea Research Institute, Nanjing Agricultural University, Nanjing, P. R. China; b Center of Cell Biology and Cancer Research, Albany Medical College, Albany, NY.

**Keywords:** caffeine, green tea polyphenols, meta-analysis, osteoporosis, tea

## Abstract

**Background:**

Previous reports have suggested a potential association of tea consumption with the risk of osteoporosis. As such association is controversial, we conducted a meta-analysis to assess the relationship between tea consumption and osteoporosis.

**Methods and Findings:**

We systematically searched PubMed, EMBASE and WanFang databases until March 30, 2016, using the keywords “tea and osteoporosis,” without limits of language. Odds ratios (ORs) with 95% confidence intervals (95% CIs) were derived by using random-effects models throughout the analyses. We conducted the analysis of the statistical heterogeneity using Cochrane *I*^*2*^. The funnel plot was used to speculate the publication bias, while the subgroup analysis and multiround elimination method were employed.

**Results:**

Our study was based on 17 journal articles, including 2 prospective cohort studies, 4 case–control studies, and 11 cross-sectional studies. In the present study, the total OR of osteoporosis for the highest versus the lowest categories of tea consumption was 0.62 (95% CI, 0.46–0.83), with significant heterogeneity among studies (*I*^*2*^ = 94%, *P* < .01). There was, however, no publication bias of the meta-analysis about tea consumption and osteoporosis. Subgroup analysis showed that tea consumption could reduce the risk of osteoporosis in all examined subgroups.

**Conclusion:**

In the present study, it can be concluded from the results that tea consumption can reduce the risk of osteoporosis.

## Abstract 2

Supplemental Digital Content is available in the text

## 1 Introduction

Osteoporosis (OP) is a pathological condition that is characterized by a bone mineral density (BMD) level of 2.5 standard deviation (SD) or more below the mean value (T-score = -2.5) for a young adult.^[1]^ It is also a metabolic bone disease that is featured by an enhanced state of bone reabsorption accompanied by diminished bone formation, leading to a reduction of BMD, deterioration of bone quality, and increasing the risk of developing fractures.^[2]^ The main clinical symptoms of OP are hip fracture, pains, physical disability, and wrist fracture. Among the aforementioned symptoms, hip fracture is the most severe consequence of OP, which leads to reduced daily activities and quality of life, even increased mortality of patients.^[3]^ OP has a heterogeneous pathogenesis, with many constitutional, lifestyle, and medical variables acting to modulate both the accretion of peak bone mass and subsequent bone loss.^[4]^ Owing to the universal public health problems associated with the increasing prevalence of OP, it is, therefore, imperative to improve upon its treatment and consequently prevent its progression.

Tea is one of the popular beverages in the world. It has been widely used in medical field because it contains antioxidants such as catechins, thearubigin, theaflavin, and other flavonoids. Tea consumption is reported to have protective effects on the cardiovascular disease, Parkinson disease, and several kinds of cancers.^[5]^ Sheng et al^[6]^ reported a nonlinear association between tea consumption and reduced risk of hip fracture. According to them, daily intake of 1 to 4 cups of tea a day reduces hip fracture.^[6]^ Green tea polyphenols (GTPs), which is extracted from green tea, is the main functional component in tea. Earlier studies have reported that GTP might improve bone density and mitigate bone loss.^[7,8]^

Several studies have been conducted on the relationship between tea intake and the risk of OP. The results from these studies are, however, inconsistent. Chen et al^[9]^ reported that habitual tea intake has a little effect on bone density and cannot significantly change the risk of fractures among postmenopausal women. Hallstrom et al^[10]^ also found that tea drinking was not associated with osteoporotic fractures. On the contrary, Hegarty et al found higher BMD in old women who drink tea, as compared with those who do not take tea. Their report, therefore, indicates that tea drinking may have a positive effect on BMD of old women.^[11]^ So far, there is no report conclusively demonstrating the relationship between tea consumption and OP. In this report, we performed a meta-analysis of prospective cohort, case–control studies, and cross-sectional studies on the purpose of investigating the relationship between tea consumption and OP.

## 2 Materials and methods

### 2.1 Literature search

An electronic search from PubMed (http://www.ncbi.nlm.nih.gov/pubmed/) and EMBASE (http://store.elsevier.com/embase) was performed up to March 30, 2016. The keywords inputted were “tea and osteoporosis,” with no language restriction. Full texts of relevant citations from all identified results were inspected and analyzed. From the main search results, relative references to the inputted key words were also searched and reviewed accordingly. Chinese Wan Fang database (http://www.wanfangdata.com.cn/) has been also searched. The study selection process was performed following the Preferred Reporting Items for Systematic Review and Meta-Analyses (PRISMA) statement.^[12]^

### 2.2 Selection criteria

All extracted reports that represented quantitative estimates regarding the linkage between tea consumption and OP were reviewed and evaluated. The reports that met the criteria set for this study were used as sample for meta-analysis in the present study. The criteria for the present study were set as follows: designs of the study are prospective cohort, case–control, and cross-sectional studies; human population studies instead of animals such as mice and rats; outcome of interest is OP; independent variables of tea consumption instead of “tea and coffee” or “caffeine”; reports on case control, and risk estimates, such as relative risks (RRs) and odds ratios (ORs) with 95% confidence intervals (CIs); and minimum of 20 cases in a report. Any report that did not satisfy such criteria was ruled out.

### 2.3 Data extraction and quality assessment

A standard data collection form was designed to arrange the extracted data of interest. Relevant aspects of extracted data of interest, with reference to name(s) of author, year of publication, design of study, sex of population, age, study period, number of case and control, category of tea consumption or OP, value of OR with 95% CI, and relative adjustments were recorded on the data collection form. The methodological quality of each study was assessed separately. The quality of data included in the present study was assessed using Newcastle–Ottawa Scale (NOS) as described by Stang,^[13]^ which was used either as a checklist or a scale. Separate NOS were, therefore, developed for prospective cohort and case–control studies.

### 2.4 Ethical statement

All results and analyses were from previous published studies; thus, no ethical approval and patient consent are required.

### 2.5 Statistical analysis

Statistical heterogeneity was analyzed using Cochrane *I*^*2*^, which depicts the percentage of variation across studies due to heterogeneity rather than chance.^[14]^ In cases where *I*^*2*^ ≥ 50% random-effects models were employed, otherwise, fix-effect models were used in the analysis. OR with 95% CI was employed in this study to measure the association between tea consumption and OP. Statistical significance was set at *P* < .05.

Subgroup analysis was conducted according to geographical location, sex, design of study, categories of OP, and study period. To explore the effect of each extracted data on the overall heterogeneity and value of OR with 95% CI, a multi-round elimination method was used in the analysis. In detail, we excluded each study included to determine the lowest heterogeneity and then we deleted it in the next round. We continued to execute the second and more rounds to rule out the relative studies until the overall heterogeneity was below 50%. Publication bias was analyzed using funnel plot. Software Review Manager 5.3 was used for the whole meta-analysis.

## 3 Results

### 3.1 Study selection

**Figure 1:**
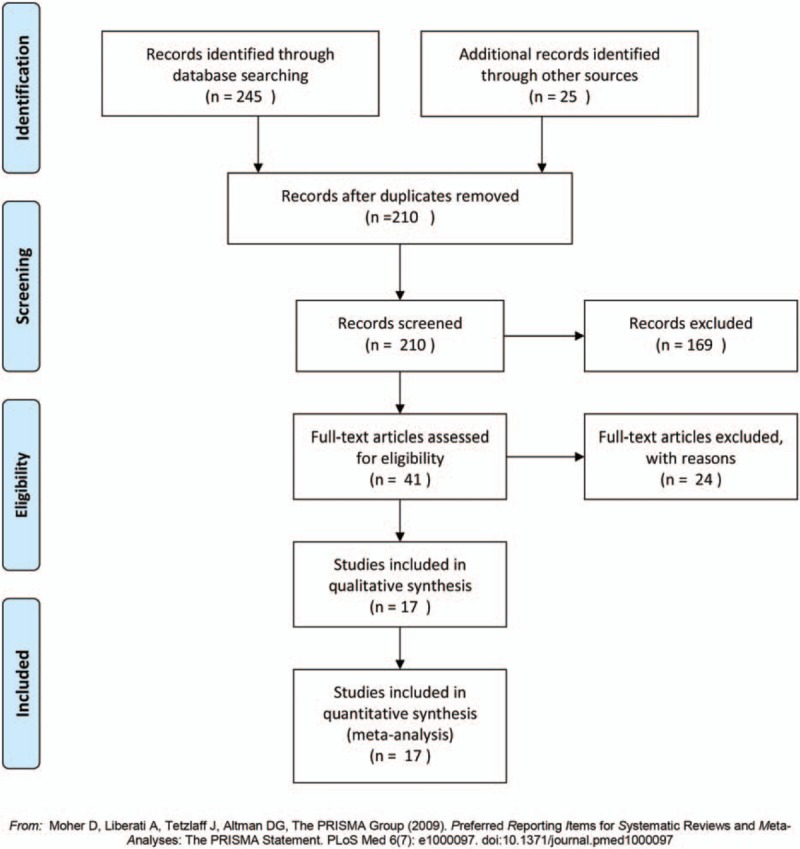
Study selection process of meta-analysis.

A flow diagram of the study selection process is shown in Fig. 1. And the specific PRISMA checklist is shown in File S1. Database search using the online search engines employed in this study led to a retrieval of 131 and 114 records from PubMed and EMBASE, respectively. From the search results, 60 duplicated records were found, and eliminated from further analysis. Upon reviewing the titles and abstracts of extracted data, studies that did not meet our criteria were removed from the sample. However, after examination of the references cited in the remaining records, 25 other records were added to the sample. After carefully reviewing the full-text of each report, we ruled out 169 reports and focused on the remaining 41 reports for further assessment. Because of the lack of the data of interest, 24 reports were dropped out. As a result, 17 references^[9,15–30]^ were selected for the meta-analysis.

### 3.2 Study characteristics

Study sample in the present study were from 4 continents: Europe, Asia, America, and Oceania. Among the 17 reports, 3 and 11 were conducted exclusively on male and female subjects, respectively. The remaining 3 were conducted on both males and females. The characteristics of the prospective studies on tea consumption and OP are presented in Table 1 . There are 2 prospective cohort studies, 4 cross-sectional studies, and 11 case–control studies among them. In the present study, the main symptom of OP was hip fracture. Ten articles that not mentioned the symptom were classified to “Ungrouped.” The study period was over 3 years in 10 studies and less than 3 years in 6 studies. One study remaining was obscure and thus classified to “Ungrouped.” The covariates mostly taken into consideration were age, body mass index (BMI), education, physical activity, alcohol drinking, and smoking. The results of quality assessment of selected studies are summarized in Table 2. Among 13 studies included, 10 were in relative high quality (over 6 stars) with an average NOS score of 7.23.

**Table 1:**
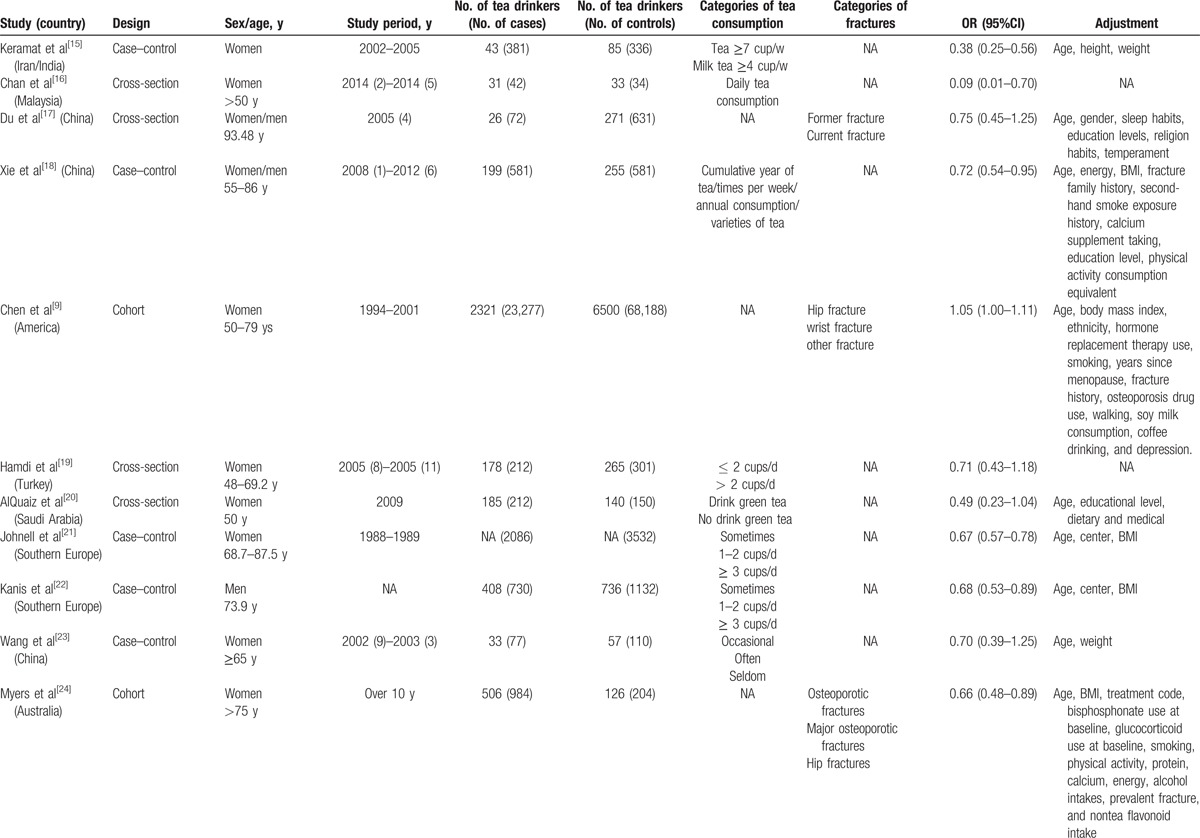
Characteristics of the prospective studies in the meta-analysis of published studies on tea consumption and the risk of osteoporosis.

**Table 1 (Continued):**
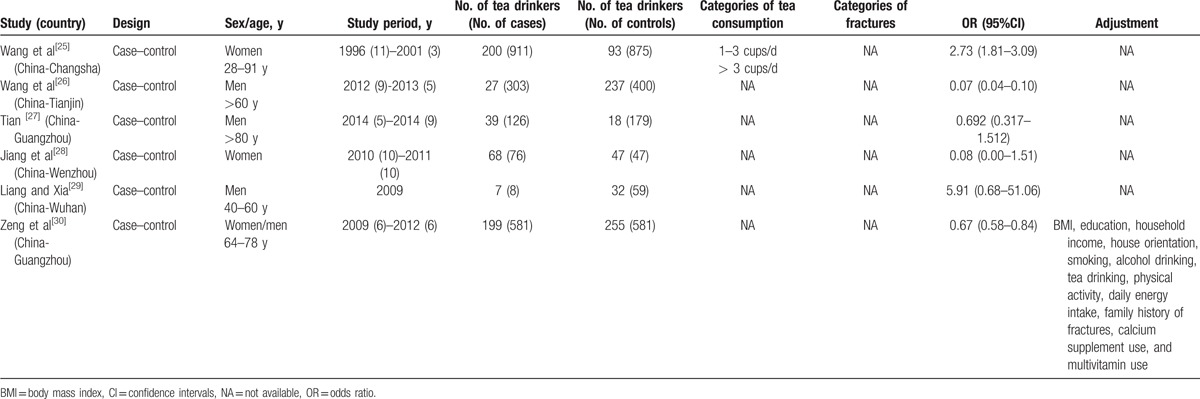
Characteristics of the prospective studies in the meta-analysis of published studies on tea consumption and the risk of osteoporosis.

**Table 2:**
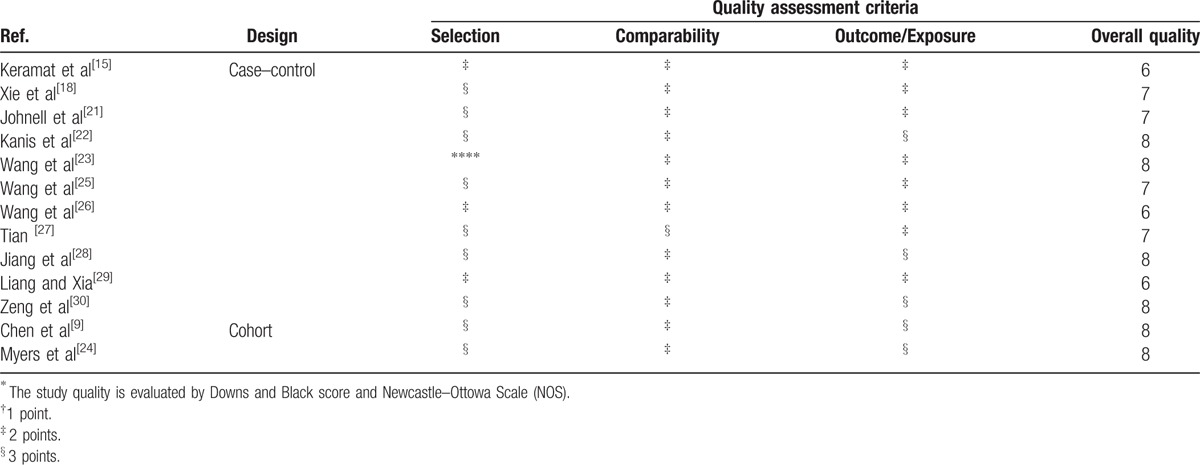
Quality score of the studies in the meta-analysis of published studies on tea consumption and the risk of osteoporosis^∗^.

### 3.3 Association between tea consumption and OP

**Figure 2:**
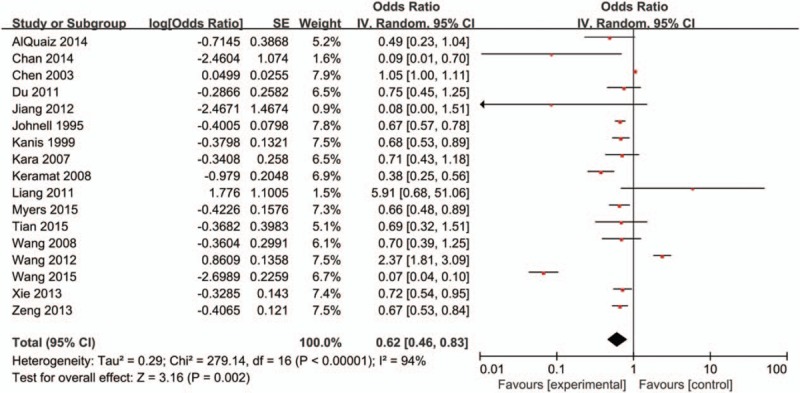
Forest plot for assessment of association between tea consumption and osteoporosis.

**Figure 3:**
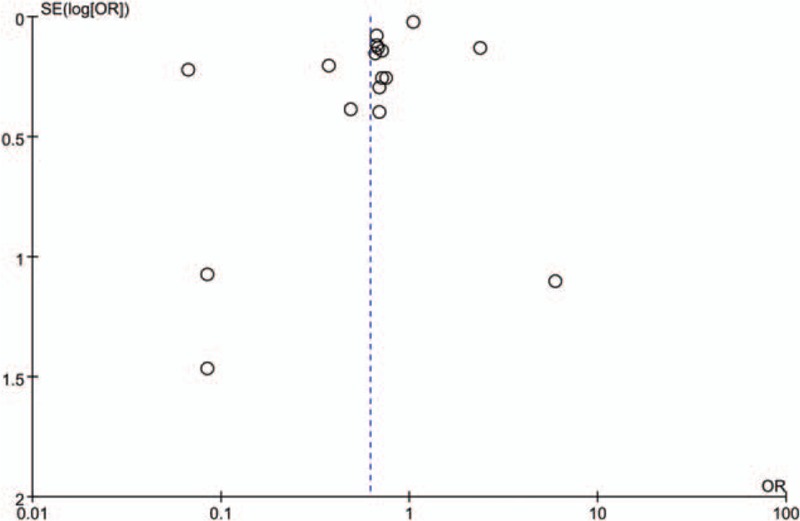
Funnel plot for assessment of publication bias.

The multivariate-adjusted ORs of each study of the highest versus the lowest tea consumption are available in Fig. 2. The total OR of OP for the highest versus the lowest categories of tea consumption was 0.62 (95% CI, 0.46–0.83), with significant heterogeneity among studies (*I*^*2*^ = 94%, *P* < .01). The assessment of publication bias for the result of the research is shown in Fig. 3. It was shown that all the studies were in a symmetrical distribution, most of which were clustered on top of the symmetric axis (Fig. 3). The result, therefore, indicated that there was no publication bias of the meta-analysis about tea consumption and OP. At relatively high heterogeneity, the reports were subjected to multiround elimination method to determine the effect of each report on heterogeneity. The result from the assessment is presented in File S2, http://links.lww.com/MD/B996. In the first round of elimination, we found that after deleting the report by Wang et al,^[26]^ heterogeneity reduced to 89%. In the second round, deletion of the report by Wang et al^[25]^ resulted in a significant reduction. Meanwhile, the ideal heterogeneity level (30%) was attained when the report by Chen et al^[9]^ was removed in the third round of the elimination process. It can, therefore, be deduced that the 3 main reports mentioned above were the main source of high heterogeneity in the sample. Even though there was a significant decrease in heterogeneity, the overall OR (95% CI, 0.57–0.74) showed no significant changes. It can also be deduced from the present study that heterogeneity and OR have no direct relationship.

### 3.4 Subgroup analyses

The reports used in the present study were classified into 5 subgroups for analysis, as summarized in Table 3. Considering the geographical location, reports from Europe (OR = 0.68, 95% CI, 0.59–0.77), Asia (OR = 0.54, 95% CI, 0.29–0.99), and Oceania (OR = 0.66, 95% CI, 0.48–0.89) had statistically significant results. Among the 3 sexual subgroups, female group (OR = 0.73, 95% CI, 0.54–0.99) showed significant decrease. Both case–control group (OR = 0.60, 95% CI, 0.37–0.97) and cross-sectional group (OR = 0.61, 95% CI, 0.40–0.94) presented significant decreasing result in 3 kinds of design of studies. When stratified by categories of OP, hip fracture (OR = 0.74, 95% CI, 0.63–0.88) was solely detected to significantly reduce the risk of OP. It was pointed that study period less than 3 years (OR = 0.46, 95% CI, 0.25–0.88) had significant association. The heterogeneity was significant when all the 17 studies were pooled in meta-analysis (*I*^*2*^ = 94%, *P* < .01). In the subgroups of geographical locations, the heterogeneity of Europe group (*I*^*2*^ = 0, *P* = .97) was very low.

**Table 3:**
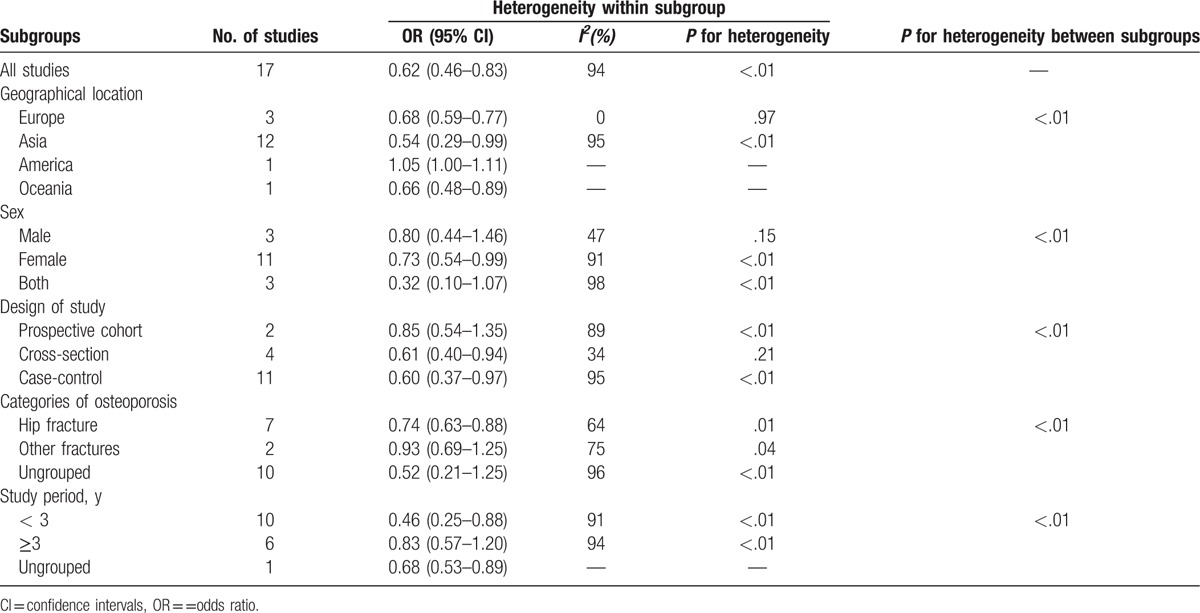
Subgroup analysis of tea consumption and osteoporosis.

## 4 Discussion

Tea is one of the popular beverages in the world. It contains antioxidants that exhibit protective impacts on human health. Tea consumption is reported to have a protective effect on the cardiovascular disease, Parkinson disease, and several kinds of cancers.^[5]^ In the present study, our analysis of 2 cohorts, 11 case–control, and 4 cross-sectional studies with 107,819 cases indicated that tea consumption may have significant statistical association with a lower risk of OP. Those case–control studies and cohort ones were all of high quality. The total OR of OP for the highest versus the lowest categories of tea consumption was 0.62 (95% CI, 0.46–0.83), with significant heterogeneity value among reports (*I*^*2*^ = 94%, *P* < .01).

Chen et al^[9]^ examined the relationship between tea intake and fracture risk in women. According to their report, tea consumption might not be associated with the risk of fracture (RR, 0.89; 95% CI, 0.78–1.06). Their result compares favorably with the result in the present study. Contrary to the results in the present study, Huang and Tang^[31]^ reported a significant association between tea drinking and osteoporotic hip/femur fractures in middle-aged and elderly men by a case–control study. Recent studies have also found that GTP is not associated with overall improvement in BMD in overweight/obese postmenopausal women.^[32]^ Therefore, more epidemiological research to assess the effect of dietary tea on OP should be conducted.

Of all the reports used in the present study, Liang and Xia^[29]^ and Wang et al^[25]^ recorded extreme OR values, 5.91 (95% CI, 0.68–51.06) and 2.73 (95% CI, 1.81–3.19), respectively. The OR value for Liang and Xia^[29]^ could be due to the fact that strong tea was used as the criterion for their studies. It is known that a cup of strong tea contains 3 to 4 g tea, which was different from the concentration of tea in other reports used in the present study. Also, the target population for their study was office workers and business men who usually consumed more strong tea. On the contrary, age of population studied (up to 91 years) may contribute to the extreme OR values reported by Wang et al.^[25]^ This is so because older people can be more vulnerable to OP due to their eating habits.

The protective effect of tea on bone may be due to its functional constituents. As an important aqueous extraction of tea, GTP was initially known to the world for its antioxidation ability via a mechanism of capturing and detoxifying reactive oxygen species (ROS). GTP can promote bone-forming osteoblast activities and inhibit the bone-resorpting osteoclast formation, which can finally result in OP. Shen et al^[33]^ provided strong evidence of GTP's bone mass conservation effect due to its antioxidant capacity, as indicated by higher liver glutathione peroxidase (GPx) activity and lower urinary 8-Hydroxy-2-deoxyguanosine (8-OHdG) level. In addition, Shen et al^[34]^ evaluated the efficacy of GTP at mitigating bone loss and microstructure deterioration, and they concluded that using antioxidant capacity of GTP can increase bone formation and suppress bone resorption, which can attenuate trabecular and cortical bone loss. Besides, Park et al^[35]^ proposed that GTP can act as a biological antioxidant in a cell cultural experimental model and protect cell from oxidative stress induced toxicity of ROS on osteoblast. Moreover, the intake of polyphenols also improved metabolic status,^[36]^ which in turn may play an indirect role in preventing OP.^[37]^

The active compounds in tea polyphenol are from a group of polyphenols called catechins, of which the main 4 monomers are (-)-epigallocatechin-3-gallate (EGCG), (-)-epigallocatechin (EGC), (-)-epicatechin-3-gallate (ECG), and (-)-epicatechin (EC). Ko et al^[38]^ found that EGC was the most effective in promoting osteogenic differentiation and increased some mRNA expression of bone formation markers runt-related transcription factors. Kamon et al^[39]^ discovered that EGCG can inhibit the osteoblast differentiation and reduce osteoclast formation in coculture of the osteoblasts from mouse newborn calvaria and mouse bone marrow cells.

Another important component of tea, caffeine, has been reported to have some adverse effects on OP, as tea polyphenols affect bone metabolism. Caffeine can inhibit activity of phosphodiesterase and then turn into agonist of adenosine cyclase and finally act on several tissues. Harris and Dawson-Hughes^[40]^ considered that daily consumption of caffeine may accelerate bone loss in women, especially in cases where the amount of caffeine is equal to or greater than that obtained from brewed coffee. According to Rapuri et al,^[41]^ the intake of caffeine above 300 mg/day can accelerate bone loss at the spine in elderly postmenopausal women. Tsuang et al^[42]^ also reported that 10 mM caffeine can significantly decrease the viability of osteoblasts in the osteoblast cultures. It could, therefore, be deduced from the aforementioned reports that caffeine can depress absorption of calcium in the duodenum, promote the excretion of calcium, and result in the loss of bone calcium. It is, however, of great interest to know that tea polyphenols and caffeine are counterparts’ contents of tea. Tea polyphenol accounts for 25% to 35% of dry tea, while caffeine accounts for 2% to 4%. This could explain why consumption of tea and caffeine, respectively, pose different effects on OP, as indicated in the results of the present study.

The results from data search using the search engines in the present study revealed that no work has been done to elucidate the relationship between tea consumption and OP by meta-analysis. This study has got several advantages; factors such as age, gender, BMI, physical activity, and education were adjusted. Moreover, subgroup analysis was also employed to evaluate the association between tea intake and OP in different subgroups. However, the present study still had several restrictions. First, this study was conducted on 17 reports with varied quality, adjustments, and sample size, and these variations may have innate influence of our concluded result. Second, due to the unconformity of categories of tea consumption in each report, among which OP fractures were chosen as classification criterion, we could not factor it in the subgroup analyses. Third, it was difficult to assess the tea intake just from a self-reported food frequency for the content of affecting factors, and so their associated errors are inescapable. And this may have an effect on the strengths of observed relationship. Fourth, as most of the evidence relies on cross-sectional and case–control studies, no conclusive casual relations can be derived. Finally, most reports used in the present study followed a case–control design that led to inherent recall and selection bias to retrospective studies. Although different kinds of reports were included, subgroup analysis was conducted to exclude any interruption.

From the subgroup analysis (Table 3), the heterogeneity in Europe group, male group, case–control study group, hip fracture, and other fractures groups decreased significantly. It can, therefore, be concluded that the heterogeneity in the present study mainly came from Asia group, female group, prospective cohort study group, and case–control study group. Moreover, in the quest to determine the exact origin of high heterogeneity by randomly picking out each report in the whole sample group, heterogeneity was observed to have dropped significantly from 94% to 30% upon removal of 3 reports using the multirounds elimination method.^[9,25,26]^ A review of the 3 reports led to a possible reason for their high heterogeneity levels. The report by Chen et al^[9]^ indicated that only hip fractures confirmed by review of medical records were used as the identification criteria in the study; other non-hip fracture data were based upon self-report, which may not be accurate. In another report, Wang et al^[25]^ focused only on females between the ages of 28 and 91 years. The large age range and single sex population may contribute to its high heterogeneity. Lastly, a review of the report from Wang et al^[26]^ revealed that the authors investigated the relationship between green tea consumption and OP. It is imperative to state that green tea alone is not representative enough for all kinds of tea, as there are more than 6 species of tea in China. It can, therefore, be speculated that the initial high heterogeneity level recorded in this study was as a result of the 3 reports aforementioned. However, the multiround elimination method employed to reduce heterogeneity level in the present study did not have any remarkable effect on the OR values with 95% CI.

From the subgroup analysis, it was observed that tea consumption can significantly reduce the risk of OP in female population, but it cannot change the situation in male population. We tried to figure out the mechanism of this result. Initially, only 3 reports were included in the “male” subgroup. The relatively small amount of reports used in this analysis may have a major effect. Then, when considering the adjustment mentioned in each report, it was realized that, among 3 reports in the “male” subgroup, Kanis et al^[22]^ regarded age, center, and BMI as its adjustment. Both Liang and Xia^[29]^ and Tian ^[27]^ did not mention the adjustment in their reports. Most of the reports have considered alcohol drinking and smoking as their adjustment. It was said that alcohol can induce the premature senescence in bone marrow derived mesenchymal stem cells to impair the osteogenic differentiation.^[43]^ In addition, smoking can also weaken bone tissue with the bad components in cigarette, among which nicotine can induce increased osteoclast differentiation and significantly increase the absorbing ability of osteoclast.^[44]^ Also, one of the mediators of the anti-osteogenic effects of cigarette smoking, the aryl hydrocarbon receptor, can adversely affect bone regeneration and bone healing.^[45]^ Thus, the benefit of tea consumption is likely to be offset significantly by these bad habits in male. This could, however, explain why tea consumption can significantly reduce the risk of OP in female population but not in males. It can, therefore, be deduced that bad living habits may reduce the effect of tea consumption on OP.

In the present, meta-analysis offered a statistical result to conclude that tea consumption reduces risk of OP. However, the exact mechanism of the relationship between tea consumption and OP still needs further research.

## Supplementary Material

Supplemental Digital Content
